# Superior Strength and Ductility of In Situ Nano TiB_2_/Al–Cu–Mg Composites by Cold Rolling and Post-Aging Treatment

**DOI:** 10.3390/ma12213626

**Published:** 2019-11-04

**Authors:** Junhui Tang, Jiwei Geng, Cunjuan Xia, Mingliang Wang, Dong Chen, Haowei Wang

**Affiliations:** 1 School of Materials Science and Engineering, Shanghai Jiao Tong University, Shanghai 200240, China; junhuitang@sjtu.edu.cn (J.T.); gengjiwei163@sjtu.edu.cn (J.G.); xiacunjuan@sjtu.edu.cn (C.X.); 2 State Key Laboratory of Metal Matrix Composites, Shanghai Jiao Tong University, Shanghai 200240, China; hwwang@sjtu.edu.cn

**Keywords:** Al composite, in situ TiB_2_ particles, mechanical properties, aging behavior, GPB zones

## Abstract

In this work, the combination of cold rolling with post-aging treatment is developed to achieve the optimal strength–ductility for the in situ nano TiB_2_/Al–Cu–Mg composite. The microstructure and mechanical properties of the composite subjected to 20% thickness reduction of cold rolling at room temperature and their evolutions upon post-aging at different temperatures were investigated by means of a tensile test, differential scanning calorimetry, scanning electron microscopy, and transmission electron microscopy. It was found that the TiB_2_ particles were effective in dislocation pinning and accumulation during the cold-rolling process. The tensile tests indicated that both the yield and ultimate tensile strengths of the cold-rolling sample increased a lot due to the dislocation strengthening and precipitation strengthening generated by dynamic precipitation during cold rolling in comparison with the conventional T6 sample. After aging at 100 °C/12 h, the elongation to failure reached ~8.4%, which was higher than the conventional T6 sample. Meanwhile, there was also a dramatic increase of strength. The yield and ultimate tensile strengths are ~644 MPa and ~726 MPa, respectively. This remarkable strength–ductility combination was due to the modified microstructure caused prior to artificial aging by the cold-rolling method and the formation of nanosized Guinier–Preston–Bagaryatsky (GPB) zones. The underlying mechanisms related to the superior strength–ductility combination were discussed regarding the microstructural characteristics in the composite.

## 1. Introduction

Strength and ductility are two of the most important mechanical properties for structural materials. However, they are often in conflict with each other. This correlation is associated with the nature of plasticity. The more difficult it is for dislocations to appear and to move, the stronger but more brittle and less ductile any crystalline material is [1]. Therefore, there is a strong push to improve the strength–ductility combination of Al-based materials for aerospace and automobile applications. The Al–Cu–Mg alloy is one of the most commonly used aerospace Al alloys due to their attractive mechanical properties [2,3,4]. Thus, it has been intensively developed to meet the advanced industrial requirement. A number of methods have been developed to improve its strengths. For example, the electropulsing treatment (EPT) is an instantaneous high-energy input method, and has been extensively applied in science and engineering fields. In recent decades, many studies have indicated that the EPT can improve the mechanical properties by the grain-refining effect [5,6,7]. Recently, severe plastic deformation (SPD) techniques (i.e., equal channel angular pressing (ECAP) [8,9], high-pressure torsion (HPT) [10,11], and accumulation-rolling bonding (ARB) [12]) have been proven as the effective approaches for microstructural refinement, leading to enhanced strength and ductility simultaneously. For instance, ECAP has been applied to different Al alloys to obtain high strength and ductility due to the higher dislocation accumulation rate in the solutionized matrix and the presence of a higher density of fine particles in the aged matrix [13]. Kim et al. [8] reported that by combining solid solution treatment, ECAP, and post-ECAP low-temperature aging, the yield strength of 2024 Al alloy was improved to >600 MPa while maintaining a respectable value of elongation to failure. However, all these methods have their own limitations such as requiring a large load, the small size of products, the high labor, and fuel expenses, degrading the possibility to be used for commercial applications. If the materials for structural applications are to be produced commercially in large quantities, a conventional processing method, such as rolling, should be more achievable. Recently, the cryogenic rolling combined with post-aging treatment has been regarded as a potential method to obtain nanostructured or ultrafine-grained Al alloys [14,15]. Normally, the low temperature (cryo) suppresses the dynamic recovery during deformation, and the cryogenic rolling can preserve a high density of defects generated by deformation, which can act as potential recrystallization sites for precipitates at relatively low strain and stress as compared to other processes. Therefore, the strength and ductility of materials can be both improved. Cheng et al. [15] reported that by combining solid solution treatment, cryo-rolling at liquid nitrogen temperature and aging at 100 °C for 100 h, the yield strength of 2024 Al alloy was increased to ~580 MPa, with a good elongation to failure of ~18%. However, this method also has weaknesses. The highly dangerous nature of carrying out a work-piece at liquid nitrogen temperature and the higher cost of its experimental setup has limited its further application. In addition, Huang et al. [16] reported that by combining solid solution treatment, conventional rolling at room temperature (to a total thickness reduction of 40%), and aging at 175 °C for 7 h, the yield strength of 2024 Al alloy reached 540 MPa. Such an attempt to obtain a high strength–ductility combination is successfully achieved by combining room temperature rolling with post-aging treatment on Al alloys [15,16,17,18].

The particle-reinforced Al matrix composites (AMCs), which possess excellent mechanical properties and high moduli, have attracted extensive investigation [19,20,21]. Recently, these composites have been applied as advanced aerospace materials. The particle-reinforced AMC is one of the developed materials with higher mechanical properties in comparison with the metallic counterpart. For example, the particulate reinforcements such as SiC [22,23,24], B_4_C [25,26], and Al_2_O_3_ [27,28] particles have been widely investigated in the Al–Cu–Mg alloy composites. In these composites, the particles are usually added by ex situ methods with the larger particle size at the microscale. Due to the poor ductility of ex situ particle-reinforced AMCs, there are rarely studies about the optimization of mechanical property using the thermomechanical treatment. Regarding the in situ particle-reinforced AMCs, the in situ TiB_2_/Al alloy composites have been successfully prepared by the salt–metal reaction route, which showed the elevated and balanced mechanical properties recently [29,30]. Geng et al. [31] performed pre-stretch aging treatment on in situ nano TiB_2_/Al–Cu–Mg composites to improve the mechanical property by tuning the microstructure. However, the improvement of strength is limited by the strain capacity introduced by the pre-stretch.

In the present work, a thermomechanical treatment (consisting of: (i) solution treatment at higher temperature to fully dissolve the second-phase particles and to produce an oversaturated solid solution; (ii) a conventional cold-rolling at room temperature to accumulate the strain; and (iii) post low-temperature aging treatment to optimize the precipitates in the matrix) was proposed to tune the mechanical properties of the in situ TiB_2_/Al–Cu–Mg composite. The effects of post-aging temperatures on both the microstructure and tensile property of the composite were focused. The underlying mechanisms for the improved mechanical property have been discussed in terms of the microstructural characteristics in the composite.

## 2. Materials and Methods

The in situ 6 wt.% TiB_2_/2024 composite with a major composition of Al–4.63Cu–1.77Mg–0.71Mn (wt.%) in matrix alloy was fabricated by the addition of a pre-weighted mixture of K_2_TiF_6_ and KBF_4_ salts into the 2024 alloy melt at 850 °C through an exothermic reaction. The melt was stirred using a home-made blade paddle mixer for 30 min, and then the slag was skimmed out completely. Afterwards, the melt was poured into a permanent mold to obtain the as-cast ingot, and the ingot was extruded at 420 °C with the extrusion ratio of 14:1 to the as-extruded state.

**Figure 1:**
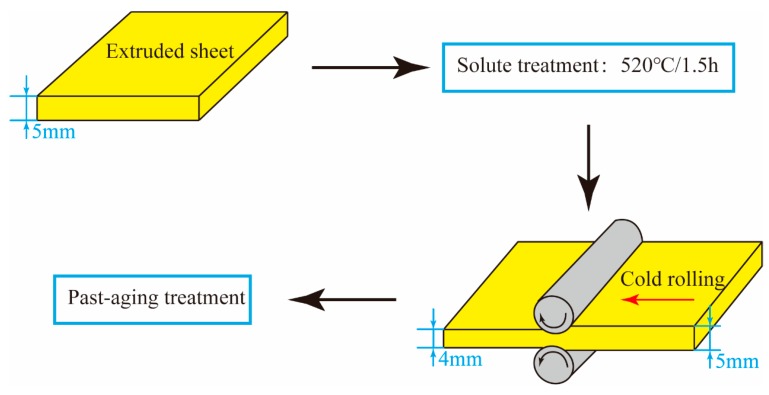
The sketch map of the experimental procedure.

The as-extruded plates with 5-mm thickness were solution-treated at 520 °C for 1.5 h followed by water quenched to room temperature. Subsequently, such samples were cold rolled (CR) at room temperature to 4 mm with a total thickness reduction of ~20%. Finally, the artificial aging treatment was carried out at 180 °C for 18 h of conventional T6 treatment and at lower temperatures (80 °C and 100 °C) for various durations to further optimize the strength–ductility combination of CR sheets. The whole procedures can be illustrated shown in as Figure 1. In order to illustrate the treated condition clearly, the description “CR@100 °C/*x*h” should represent “CR + aging at 100 °C/*x*h”.

The tensile test was performed on a Zwick/Roell Z020 testing machine (Zwick, Ulm, Germany) at a strain rate of 6 × 10^−4^ s^−1^. All the tensile samples with the gauge size of 54.5 mm length × 15 mm width were machined along the rolling direction. The tensile properties of the composites were calculated by averaging the values of three parallel samples. The differential scanning calorimetry (DSC, 204F1) investigations were performed in the flowing Ar atmosphere at a constant heating rate of 10 °C/min with the polished composite disks of 5-mm diameter sealed in Al pans. The observed samples were prepared by metallographic grinding and polishing paralleled to the rolling direction. The microstructures were examined by scanning electron microscopy (SEM, TESCAN MAIAS XMU, TESCAN, Brno, Czech Republic). The transmission electron microscopy (TEM, FEI Talos F200X, Hillsboro, OR, USA) observations were performed to investigate the microstructure evolution during aging. All the TEM facilities were operated at 200 kV. The TEM thin foil of the 3-mm diameter disk was prepared by metallographic grinding and polishing, followed by the twin-jet electropolishing using a mixture of 25 vol% nitric acid and 75 vol% methanol electrolyte. The dislocation density was examined by the Ultima IV X-Ray diffractometer with Cu Kα radiation (λ = 0.1542 nm). The range of diffraction angle was from 110° to 120°, and the scan speed was 2°/min. As a measure of peak broadening, the true full-width at half-maximum (FWHM) of the peak was obtained by deducting the instrumental broadening.

## 3. Results and Discussions

**Figure 2:**
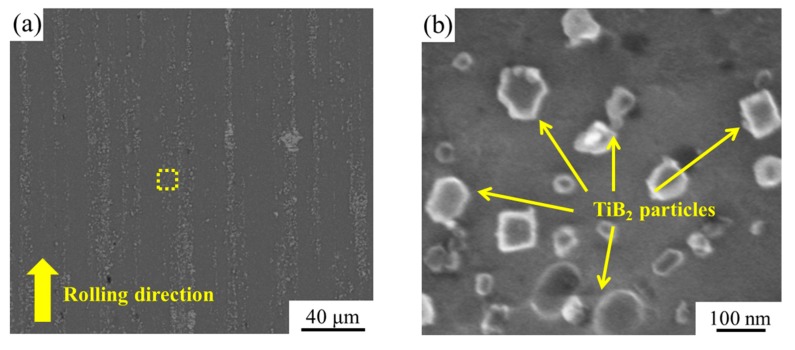
SEM (backscattered electron) micrographs of an in situ TiB_2_/2024 composite (**a**) low magnification image along the rolling direction and (**b**) corresponding high magnification image of the zone boxed in (**a**).

Figure 2a,b shows the SEM (backscattered electron) micrographs of the CR-treated TiB_2_/2024 composite under low and high magnification, respectively. In Figure 2a, the rolling direction is indicated by the arrow. The matrix is Al alloy, and the particles are TiB_2_. It is noticeable that some particles in the composites are agglomerated to form the clusters, but there are sparse TiB_2_ particles in the other regions. The TiB_2_ particles have exhibited the paralleling bands along the unidirectionally rolling direction. From Figure 2b, it is obvious that the sizes of TiB_2_ particles are mainly in <100 nm.

**Figure 3:**
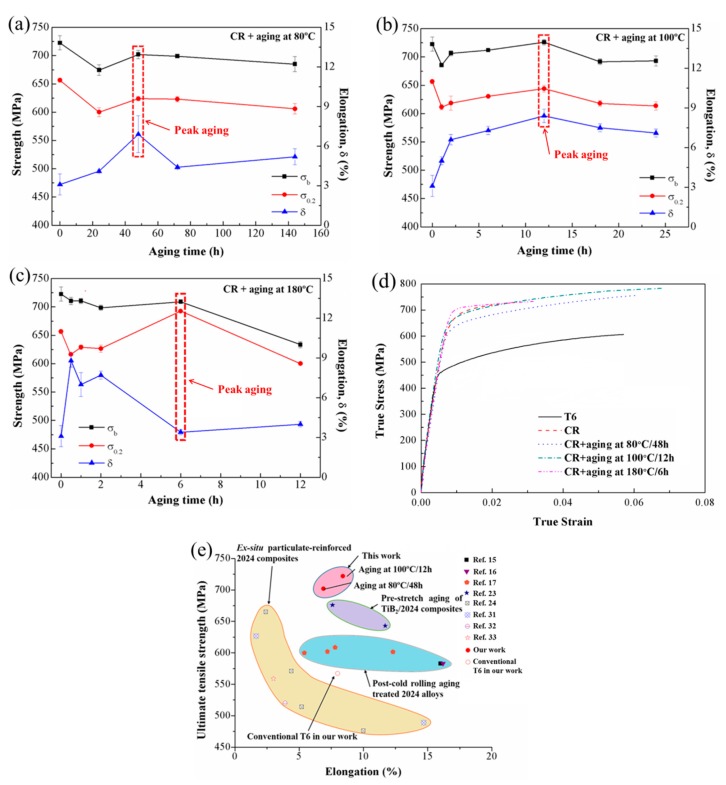
Tensile properties of in situ TiB_2_/2024 composite versus aging time of (**a**) 80 °C, (**b**) 100 °C, and (**c**) 180 °C, correspondingly (the red boxes indicate the peak aging of the given aging temperature). (**d**) True stress–strain curves of TiB_2_/2024 composites under different conditions. (**e**) Tensile properties of the TiB_2_/2024 composite in this work compared with the available literature data of 2024 alloys and particle-reinforced 2024 composites [15,16,17,23,24,31,32,33].

Figure 3a–c shows the tensile properties of the CR composites aged at 80 °C, 100 °C, and 180 °C, accordingly. For the CR composites aged at 80 °C and 100 °C, the strengths decrease initially due to the recovery in the matrix, and then increase with the precipitation of Guinier–Preston–Bagaryatsky (GPB) zones until the peak aging. After the peak aging state, the effect of dislocation density loss exceeds the precipitation of the GPB zones; thus, the strength decreases thereby. For the CR composites aged at 180 °C, the initially loss of strengths is also because of the recovery. Then, due to the precipitation of *S* phases, the strengths reach peak aging. With the coarsening of the precipitates, the strength decreases accordingly. The times to reach peak aging (48 h for 80 °C, 12 h for 100 °C, and 6 h for 180 °C (highlighted by the red boxes in Figure 3a–c)) are reduced with the increasing aging temperature. The shorter time to reach peak aging with the increased aging temperature could be attributed to the formation of high-density dislocations and vacancies introduced by the cold-rolling process. Therefore, the heterogeneous nucleation of precipitates is accelerated, and there is a short-circuit diffusion path for solute atoms speeding up the growth of precipitates.

The tensile properties and the corresponding true stress–strain curves of the in situ TiB_2_/2024 composite with different post-aging treatments are shown in Table 1 and Figure 3d. The composite in the T6 state has relatively low strength and ductility, and its yield strength (σ_YS_), ultimate tensile strength (σ_UTS_), and elongation (δ) are ~459 MPa, ~567 MPa, and ~7.5%. After cold rolling at room temperature with the thickness reduction of 20%, the σ_YS_ (σ_YS_ = ~653 MPa) is 42% higher, and the σ_UTS_ of the CR sample (σ_UTS_ = ~710 MPa) is 25% higher than the T6 sample. However, its δ is only ~3.9%, which is typical for cold-worked metals. The increased strength and decreased ductility of the CR sample is attributed to work hardening caused by cold-rolling.

Due to the low aging temperature, the variation of tensile properties under aging at 80 °C is slight. On the one hand, the σ_YS_ and σ_UTS_ of CR@80 °C/48 h sample are ~624 MPa and ~702 MPa, respectively, which are only 4% lower and 1% lower than the σ_YS_ and σ_UTS_ of the CR sample. On the other hand, its δ is elevated to ~6.9%. Increasing the aging temperature, the σ_YS_ and σ_UTS_ of the CR@100 °C/12 h sample are ~644 MPa and ~726 MPa, respectively. Remarkably, the δ increases from ~3.9% to 8.4%, which is higher by a factor of 1.2 than the CR sample. When the aging temperature is increased to 180 °C, the σ_YS_ of the CR@180 °C/6 h sample reaches a remarkable ~693 MPa, and the σ_UTS_ is ~709 MPa. However, the δ decreases to only ~3.2%. Conclusively, comparing with the CR@80 °C/48 h sample and the CR@180 °C/6 h sample, the CR@100 °C/12 h sample shows superior combined mechanical properties.

The decreased σ_YS_ of the CR@80 °C/48 h sample is due to the recovery of the generated dislocations by deformation but relatively limited precipitations in the post-aging process. For the CR@180 °C/6 h sample, a large amount of *S* phases (Al_2_CuMg) precipitated during the post-aging process can not only compensate for the loss of strength caused by recovery of dislocations, but further increase the σ_YS_ by ~40 MPa compared with the CR sample. However, because of the coarsening of precipitates, the ductility deceased to only ~3.2%. For the CR@100 °C/12 h sample, the improved ductility should be attributed to the relaxation of internal stress during post-aging processing. This process is in favor of the dislocation accumulation before saturation under tensile stress [4]. Besides, the elongation to failure is increased, and the uniform elongation is also improved, as shown Figure 3d. Compared with the CR sample whose uniform elongation is ~3.2%, the CR@100 °C/12 h sample owns a higher uniform elongation of ~7.1% due to the precipitation of nanosized GPB (Guinier–Preston–Bagaryatsky) zones [3,18]. Therefore, the higher ductility of the CR@100 °C/12 h sample can be explained.

**Figure 4:**
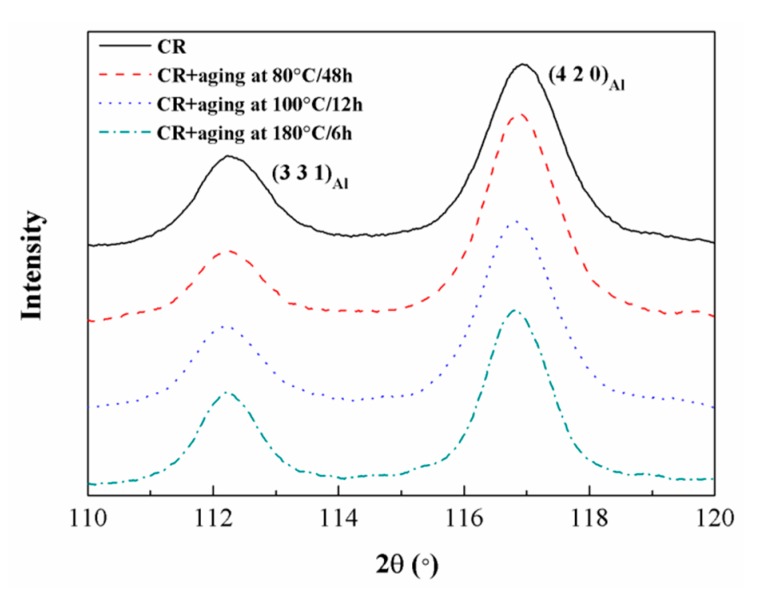
XRD patterns of the TiB_2_/2024 composite in different states.

In Figure 4, the variations of dislocations densities for both the CR sample and CR samples with different post-aging treatments are presented by XRD (X-ray diffraction spectrum) patterns. The FWHMs of Al(3 3 1) and Al(4 2 0) peaks narrow down from 1.076° and 1.136° to 1.049° and 1.118° after aging at 100 °C/12 h, accordingly (Table 2). There are mainly two reasons for the narrowing of the Al peaks in the composites [34], which are grain refinement and dislocations multiplication or annihilation. After solution treatment and the cold-rolling process, the grain size of the composites remains stable and almost unchanged, since the temperature is not enough to make it grow up in the composite [35]. Therefore, the variations of the FWHMs at both peaks mainly demonstrate the change of dislocation densities.

When the solid solution-treated (SST) samples are cold rolled with the thickness reduction of 20%, a lot of dislocations are introduced in the CR sample. After the aging of the CR sample, the dislocation densities should decrease due to the recovery, which explains the higher elongation and the lower σ_YS_ of the CR@100 °C/12 h sample compared with the CR sample. For the CR@80 °C/48 h sample, the FWHMs of the Al(3 3 1) and Al(4 2 0) peaks narrow down to 1.035° and 1.081° (Table 2). Although the aging temperature is low, the recovery of dislocations is more than that of the CR@100 °C/12 h sample due to the long aging time. Meanwhile, the decreased σ_YS_ is not only attributed to the recovery of dislocations, but also to the few GPB zones precipitated under low aging temperature. When the CR sample is aged at 180 °C for 6 h, the FWHMs of Al(3 3 1) and Al(4 2 0) peaks narrow down to 0.958° and 0.995°. Although the dislocation recovery is much more than the CR@100 °C/12 h sample, the improved σ_YS_ is mainly because of large amounts of *S* phase precipitates.

**Figure 5:**
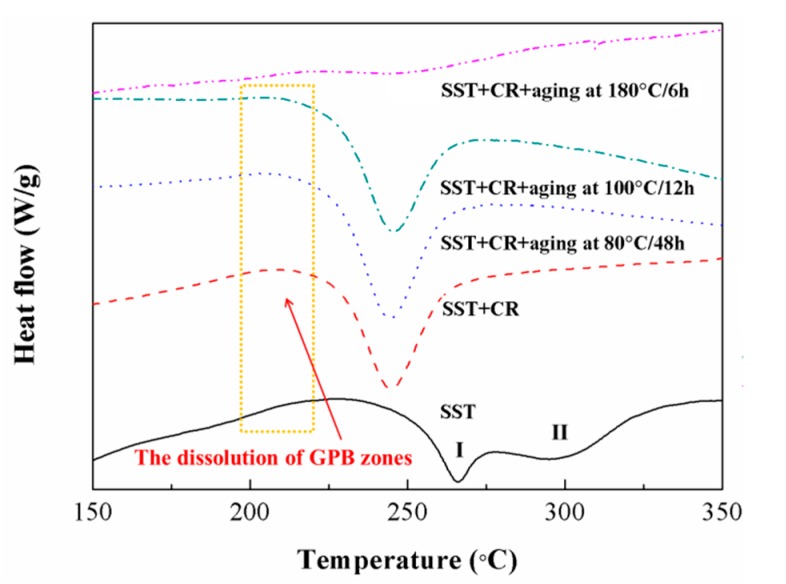
Differential scanning calorimetry (DSC) curves of TiB_2_/2024 composite in different states.

Figure 5 presents the DSC curves of the composites experiencing different aging treatment. For the SST sample, a strong exothermic effect containing two overlapping peaks (I and II) appears at about 200–350 °C. Peak I is due to the formation of *S*-type precipitates, whereas Peak II is associated with the formation of type II *S* phases. The type II *S* phase has a weaker effect on strength because of its relatively larger size [4]. An endothermic effect of peak in the frame at about 170–240 °C is about the dissolution of GPB zones. In the *SST* + CR sample, only one exothermic peak exists between 200 °C and 350 °C. Compared with the *SST* sample, the exothermic peak of *S* precipitates in the composites is sharper and shifts to a much lower temperature range. It has suggested that the precipitation kinetics of *S* phases in the CR sample is enhanced due to the dislocations generated during the cold-rolling process providing nucleation sites for *S* precipitates and atomic diffusion paths [36]. Comparing with the CR sample, the exothermic peaks of the CR@80 °C/48 h sample and the CR@100 °C/12 h sample are quite similar to the exothermic peak of the CR sample as a result of relatively few amounts of *S* phases precipitated under the low aging temperature. The exothermic peak of the CR@180 °C/6 h sample disappeared due to the precipitation of *S* phases during the aging process.

Conclusively, after post-cold rolling aging treatment at 100 °C for 12 h, a good combination of strength and ductility can be achieved. In comparison with other post-cold rolling alloys, a pre-stretched aging in situ TiB_2_/2024 composite and ex situ 2024 composites (Figure 3e), the post-cold rolling TiB_2_/2024 composite has exhibited better combined mechanical properties.

**Figure 6:**
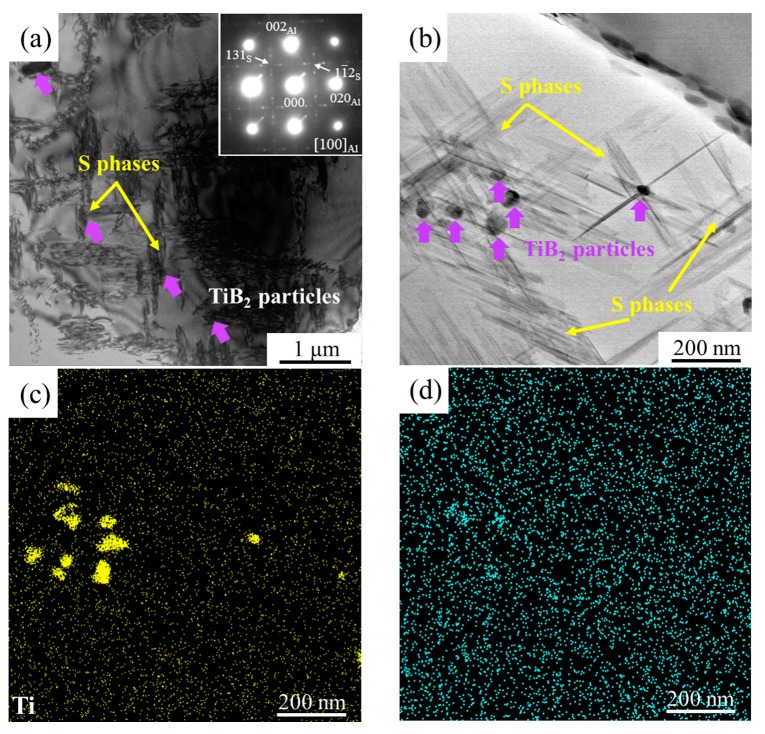
TEM images showing the morphology of *S* precipitates in TiB_2_/2024 composites under the T6 condition: (**a**,**b**) bright-field (BF) image, (**c**,**d**) corresponding element mapping. Inset in (**a**) is the corresponding selected area electron diffraction (SAED) patterns recorded along <001>_Al_.

The TEM micrographs of TiB_2_/2024 composites in their T6 states are exhibited in Figure 6a–d. Figure 6a shows that the *S* phases precipitate preferentially on dislocations around TiB_2_ particles after the conventional T6 treatment. The morphology of the needle-like *S* precipitates (length larger than 200 nm) that aggregated near TiB_2_ particles are clear at a higher magnification (Figure 6b). The distribution of TiB_2_ particles is confirmed by corresponding element mapping (Figure 6c,d). The precipitation of *S* phases is responsible for the peak aging hardening. The matrix near TiB_2_ particles can be regarded as the precipitation-accelerated zones, while the other regions with few TiB_2_ particles form precipitation-free zones. Although the strength reaches the maximum value, the distribution of *S* precipitates is still very inhomogeneous. Since the precipitation-free zones are softer than the precipitated matrix, the plastic flow should occur preferentially within these regions [37,38,39,40]. Thus, both the strength and ductility of TiB_2_/2024 composites are adversely influenced by precipitation-free zones because of their vulnerability to premature fracture. The precipitation behavior under the T6 condition has confirmed that the inhomogeneous distribution of *S* precipitates accompanying the precipitation-free zones are ineffective at improving the comprehensive mechanical properties of TiB_2_/2024 composites.

**Figure 7:**
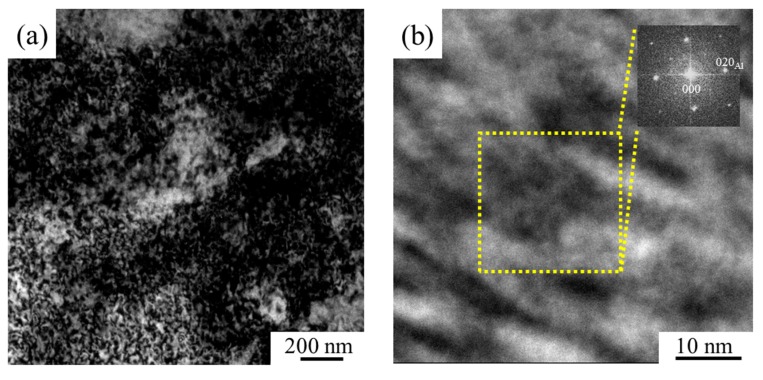
Microstructure of TiB_2_/2024 composites after cold rolling: (**a**) BF TEM image and (**b**) high-resolution TEM (HRTEM) image. Inset in (**b**) is the corresponding fast Fourier transform (FFT) pattern recorded along <0 0 1>_Al_.

The microstructure of TiB_2_/2024 composites after cold rolling was examined using TEM. The BF TEM and high-resolution TEM (HRTEM) images along a zone axis <001>_Al_ and the corresponding fast Fourier transform (FFT) pattern are presented in Figure 7. From Figure 7a, a large number of homogeneous distributed dislocations are effectively pinned and accumulated due to the presence of TiB_2_ particles after cold rolling, which are in favor of the uniform deformation and reducing local stress concentration during the tensile test. The corresponding HRTEM image is shown in Figure 7b. According to the FFT pattern, the nanoprecipitates are identified by the streaking of the diffraction spots. Due to the difference from the reported diffraction patterns of GPB zones, *S’’*, and *S/S’* phase with 12 equivalent variants [3], the very fine precipitates are considered to be solute clusters created by dynamic precipitation during cold rolling at room temperature. The increase in strength of the CR sample is mainly because of the work-hardening mechanism generated by a large number of dislocations. Aside from this point, the precipitation of very fine solute clusters also plays an important role for the enhanced strength of the precipitation-strengthening mechanism in this study. Thus, there is an σ_YS_ increase of ~194 MPa compared to the T6 sample. However, the strain hardening is known to have an adverse effect on the ductility. During the cold rolling, the composite undergoes large plastic deformation, and thus the dislocation density increases significantly. These tangled dislocations are difficult to slip, decreasing the ductility of the composite. So, the decrease of ductility from ~7.5% to 3.9% can be interpreted.

**Figure 8:**
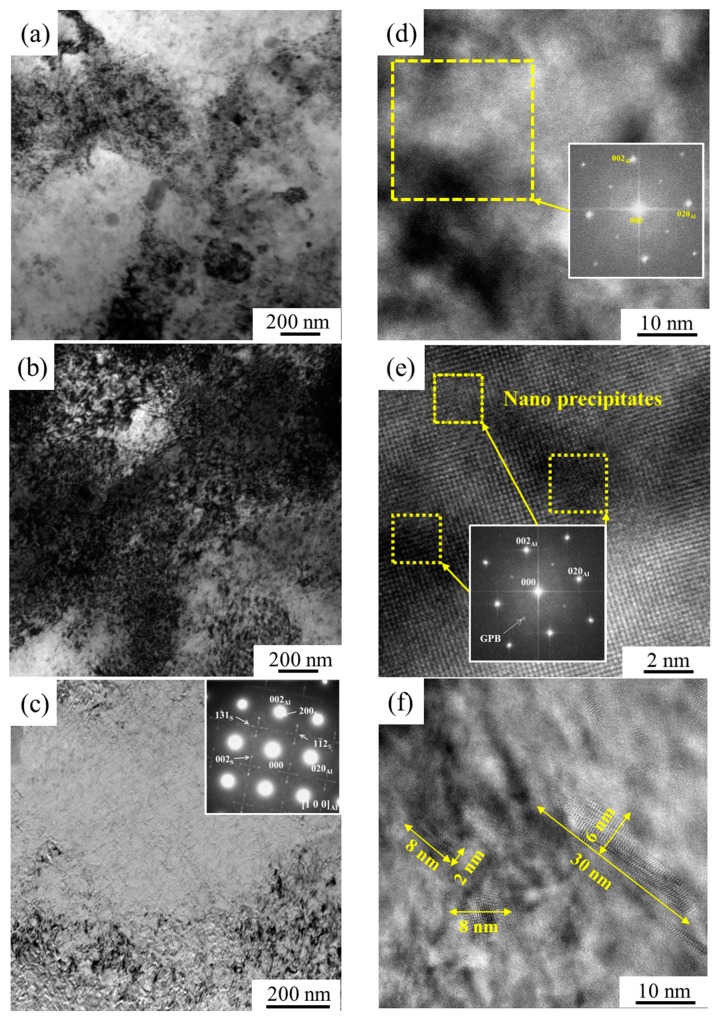
TEM micrographs of the cold-rolled (CR) sample under different post-aging conditions: (**a**) CR@80 °C/48 h, (**b**) CR@100 °C/12 h, (**c**) CR@180 °C/6 h; HRTEM images: (**d**,**e**) showing uniformly distributed nanosized precipitation regarding (**a**,**b**,**f**) showing *S* precipitates regarding (**e**). Insets in (**c**–**e**) are the corresponding SAED and FFT patterns.

Figure 8 shows the TEM micrographs of the CR sample under different post-aging conditions. During the low-temperature aging process, two microstructural changes are formed as shown in Figure 4 and Figure 8a,b, including:

(i) During post-aging, the reduction of dislocation density due to the recovery can proceed simultaneously with the *S*-phase precipitation. This also has been considered to cause the decrease of the strength in the CR samples [5].
(ii) Since the dislocation density increases drastically during the cold rolling, which provides heterogeneous nucleation sites for precipitation, there is an increasing density of nanosized precipitates in comparison with T6 treatment. For the CR@80 °C/4 h sample, although the aging temperature is quite low, the long aging time (48 h) has resulted in the more reduced dislocation density comparing with the CR@100 °C/12 h sample. Besides, there are relatively few nanosized precipitates (Figure 8d) due to the low aging temperature. Thus, the ~29 MPa loss of σ_YS_ compared with the CR sample can be explained. For the CR@100 °C/12 h sample, although the density of dislocations decreases, the precipitation of nanosized precipitates is noticeable and is rarely coarsening (Figure 8e). The strength of the material depends on the interaction of the second phase particles and mobile dislocations. Fine precipitates increase the strength of the material due to the Orowan strengthening effect [41]. On one hand, the contribution to strength from precipitation can compensate for the loss of strength causing from the reduced dislocations density, although there is still a ~9 MPa loss of σ_YS_. On the other hand, the reduced density of dislocations and homogeneously distributed nanosized precipitates contribute to the dramatic improvement of δ from ~3.9% to 8.4% (Table 1).

Comparatively, the CR@180 °C/6 h sample shows the lower density of dislocations and higher density of *S* precipitates according to Figure 4 and Figure 8c, respectively. Even though the size of the *S* precipitates are coarsened compared to the CR@100 °C/12 h sample, they are still in a fine nanoscale (~3–8 nm in width and ~12–30 nm in length) in the CR@180 °C/6 h sample. It is clear that the contribution to strength from precipitation not only compensates for the loss of strength due to the dislocation density decrease, but also further increases the yield strength by ~40 MPa as a result of the effectiveness of a high density of *S* precipitates in the strength improvement. Although the dislocation density introduced by cold rolling is decreased via recovery, which can increase δ, a high density of *S* precipitates should increase the strain concentration to some extent. The *S* precipitates are found to be effective in pinning dislocation and resisting the gliding of dislocation, which can impair the ability of further plastic deformation during a tensile test. It is likely that the improvement from recovery is not enough to compensate for the decrease in ductility caused by a large amount of *S* precipitates. Thus, the δ decreases to 3.2% in the CR@180 °C/6 h sample (Table 1).

**Figure 9:**
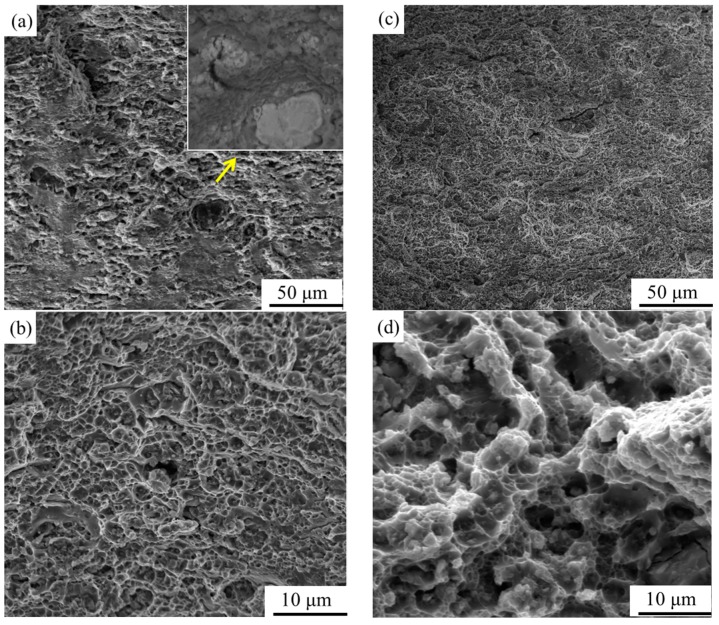
Tensile fracture morphologies of specimens under different post-aging conditions: (**a**) CR, (**c**) CR@100 °C/12 h; (**b**,**d**) are high magnification images. The inset in (**a**) is a high-magnification backscattered electron image of zone pointed by arrow.

Figure 9 shows the SEM images of the tensile fracture morphologies of the materials under different post-aging conditions. The CR sample presents brittle fracture features composed of fewer and finer dimples, and the dimple depth is much shallower (Figure 9a,b). By contrast, the CR@100 °C/12 h sample exhibits a typical ductile fracture features with many large and deep dimples (Figure 9c,d). Thus, the better ductility of the CR@100 °C/12 h sample can be explained. In addition, some larger intermetallic particles that acted as microcrack precursors can be observed on the fracture morphologies of the CR sample (Figure 9a), which are harmful to its ductility.

## 4. Conclusions

By combining the cold rolling with the suitable post-aging treatment (aging at 120 °C for 12 h), a good strength–ductility combination can be achieved in the in situ TiB_2_/2024 composites (σ_YS_ = ~644 MPa, σ_UTS_ = ~726 MPa, δ = ~8.4%).

The uniform nucleation sites have been significantly increased due to the high density of dislocations introduced by the cold-rolling process. The uniformly distributed nanosized GPB zones significantly improve the uniform plastic deformation of composites, together with the accumulated dislocations responsible for the enhancement of strength. The strengthening mechanism for the peak aging treatment of the CR sample should depend on the dislocation strengthening due to the high density of dislocations introduced by cold rolling, and the precipitation strengthening from the very fine GPB zones in the composite.

The conventional cold-rolling process and post-aging treatment employed in this study could be easily adapted to the current industrial processes and avoid the time-consuming heat treatments. Thus, the potential for large-scale aerospace industrial applications should be expected.

## Figures and Tables

**Table 1:** Tensile properties of TiB_2_/2024 composites under different conditions.

Conditions	Yield Strength (σ_YS_, MPa)	Ultimate Tensile Strength (σ_UTS_, MPa)	Elongation (%)	Uniform Elongation (%)
T6	459 ± 4	567 ± 3	7.5 ± 0.2	6.2 ± 0.1
CR	653 ± 4	710 ± 12	3.9 ± 0.8	3.2 ± 0.5
CR@80 °C/48 h	624 ± 4	702 ± 8	6.9 ± 1.4	5.0 ± 0.9
CR@100 °C/12 h	644 ± 6	726 ± 5	8.4 ± 0.5	7.1 ± 0.2
CR@180 °C/6 h	693 ± 1	709 ± 1	3.2 ± 0.2	2.8 ± 0.5

**Table 2:** The full-width at half-maximums (FWHMs) of Al(3 3 1) and Al(4 2 0) peaks for the TiB_2_/2024 composite in different states. CR: cold rolled.

Conditions	FWHMs of Al(3 3 1) Peak	FWHMs of Al(3 3 1) Peak
CR	1.076°	1.136°
CR@80 °C/48 h	1.035°	1.081°
CR@100 °C/12 h	1.049°	1.118°
CR@180 °C/6 h	0.958°	0.995°
